# Randomized pilot trial of intraoperative hemoadsorption in clopidogrel treated patients undergoing urgent coronary artery bypass grafting

**DOI:** 10.1186/s13019-026-04607-7

**Published:** 2026-07-27

**Authors:** Alireza Jahangirifard, Seyed Bashir Mirtajani, Is´haq Al-Aamri, Kumait Al-Lawati, Mandana Hosseinzadeh, Daniel Wendt, Efthymios N. Deliargyris, Mohammad Hossein Mandegar

**Affiliations:** 1 National Heart Center, Royal Hospital, Muscat, Oman; 2 Chronic Respiratory Disease Research Center, National Research Institute of Tuberculosis and Lung Diseases (NRITLD), Shahid Beheshti University of Medical Sciences, Tehran, Iran; 3 Cardiac anesthesia consultant, National Heart Centre, Royal hospital, Muscat, Oman; 4 McMaster University, Hamilton, ON Canada; 5 CytoSorbents Europe GmbH, Müggelseedamm 131, 12587 Berlin, Berlin, Germany; 6 University Hospital Essen, Hufelandstr. 55, 45141 Essen, Germany; 7 CytoSorbents Inc, 305 College Road, Princeton, NJ 08540 USA; 8 Department of Cardiac Surgery, Shariati Hospital, Tehran University of Medical Sciences, Tehran, Iran

**Keywords:** Hemoadsorption, CABG, Clopidogrel, Bleeding, CytoSorb, P2Y_12_ inhibitor

## Abstract

**Background:**

Patients receiving clopidogrel who undergo urgent CABG before the recommended 5-day washout period are at increased risk of perioperative bleeding. This study evaluated whether intraoperative hemoadsorption can reduce bleeding in such patients.

**Methods:**

Patients undergoing CABG within 24 h of clopidogrel discontinuation were randomized 1:1 to intraoperative hemoadsorption (Group 1) or control (Group 2). In the intervention group, the device was incorporated into the cardiopulmonary bypass (CPB) circuit. Efficacy was assessed by 24-hour chest tube drainage (CTD), blood product transfusions and surgical revisions for bleeding control.

**Results:**

A total of 50 patients were randomized (mean age 64 ± 8.6 years, 82% male). There were no differences in baseline characteristics between groups. Group 1 showed significantly reduced median 24-hr CTD of 300mL [250–400] vs. 500mL [400–700], *p* = 0.0003, transfusions of packed red blood cells 0 [0–1] vs. 1 [0–2] units, *p* = 0.047), fresh frozen plasma 0 [0–0] vs. 2 [2–3] units, *p* < 0.0001) and platelets 0 [0–0] vs. 2 [1–3] units, *p* < 0.0001 compared with Group 2. One revision for bleeding control occurred in Group 2 (4.0%). No device-related serious adverse events were observed.

**Conclusion:**

In this cohort of clopidogrel-treated patients undergoing CABG within 24 h of discontinuation, intraoperative hemoadsorption was associated with lower postoperative bleeding and transfusion requirements.

## **Background**

Coronary revascularization remains the cornerstone of acute coronary syndrome (ACS) treatment with approximately 10% of patients requiring coronary artery bypass grafting (CABG). Guideline recommended dual antiplatelet therapy plays a central role in ACS management; however, it poses significant challenges in patients undergoing surgical revascularization due to an elevated risk of perioperative bleeding [1, 2].

Clopidogrel, an oral thienopyridine antiplatelet agent, irreversibly inhibits the P2Y_12_ receptor on the ADP (adenosine diphosphate) chemoreceptor of platelet membranes [3]. As a prodrug, it requires hepatic activation by cytochrome P450 enzymes to exert its therapeutic effect [4]. Clopidogrel is approved for the treatment of stable ischemic heart disease and ACS [3–6], and it is widely used in the long-term management of ischemic stroke and peripheral vascular disease (PVD) [7–12]. The most common side effect of clopidogrel is bleeding, which is exacerbated when used concomitantly with aspirin or other anticoagulants. This risk becomes more clinically significant in the context of major surgeries, such as cardiac procedures, where it may increase postoperative morbidity and mortality [11].

Despite the implementation of various perioperative strategies to minimize bleeding, substantial bleeding may still occur, necessitating the transfusion of blood products or synthetic coagulation factors. However, the clinical benefit of transfusion therapy remains controversial, with several studies linking transfusions to increased complications, prolonged ICU and hospital stays, higher healthcare costs, and increased mortality [13–15]. Thus, developing effective strategies to reduce bleeding is of paramount importance.

A sorbent-filled hemoadsorption cartridge, CytoSorb (CytoSorbents Inc., Princeton, NJ, USA) has emerged as a novel intraoperative intervention [16]. This device has been shown to remove the reversible P2Y_12_ inhibitor ticagrelor and direct-oral anticoagulants (DOACs) from blood. Early reports with intraoperative device use in clinical practice have shown reductions in perioperative bleeding in patients on ticagrelor, apixaban and rivaroxaban undergoing urgent cardiac surgery [17–22]. Of note, ticagrelor binds reversibly to platelets and as such can be removed from circulation by an intraoperative device during on-pump CABG surgery, while clopidogrel and prasugrel that bind irreversibly to platelets do not allow that option. Accordingly, CytoSorb is CE mark approved for ticagrelor and rivaroxaban removal during on-pump cardiac surgery [23].

To date, it is unclear whether the use of the device can also reduce perioperative bleeding in patients on clopidogrel or prasugrel since the active metabolites of these agents bind irreversibly to platelets. Currently, only one pilot study has evaluated device performance in patients on clopidogrel [21]. Therefore, the aim of this randomized controlled trial was to assess the effectiveness of intraoperative hemoadsorption in reducing perioperative bleeding in patients on clopidogrel undergoing urgent CABG within 24 h of drug discontinuation.

## Materials and methods

### Study design

This randomized clinical trial was approved by the Biomedical Research Ethics Committee of Shahid Beheshti University of Medical Sciences, at Masih Daneshvari Hospital in Tehran (IR.SBMU.NRITLD.REC.1402.246). The trial was registered at the Iranian Registry of Clinical Trials (IRCT Code: IRCT20150725023332N7). Informed consent was obtained prior to randomization.

## Patient selection and treatment

**Fig. 1:**
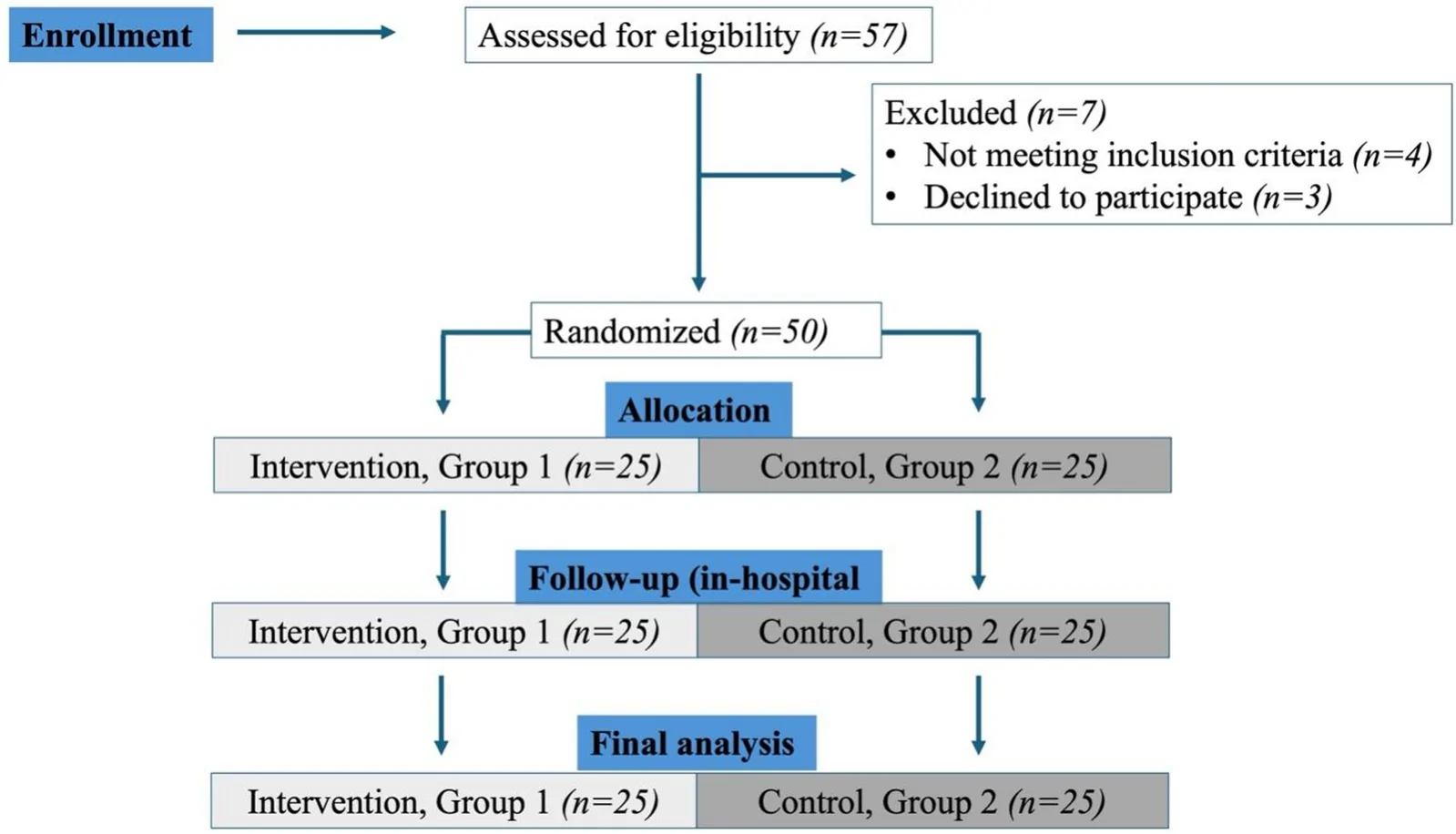
CONSORT enrolment flow-chart

The intended population comprised of adult ACS patients on DAPT requiring urgent on-pump CABG within 24 h of clopidogrel discontinuation. None of the patients had an additional washout period. Exclusions included abnormal coagulation profile, end-stage renal disease or current need for renal replacement therapy, liver failure, left ventricular ejection fraction (LVEF) < 30%, patients requiring intra-aortic balloon pump (IABP) or extracorporeal membrane oxygenation (ECMO), concurrent treatment with immunosuppressive agents (excluding corticosteroids), or severe immunosuppression. A total of 57 patients were screened. Eligible patients were randomly assigned in a 1:1 ratio to the hemoadsorption or control group using a random number table generated by an independent individual not involved in patient care, outcome assessment, or data analysis. Patients were blinded to treatment allocation at the time of information and consent. Furthermore, outcome assessors and statistical analysts were blinded to group assignment through the use of coded study data. Figure 1 shows the CONSORT 2025 enrolment flow diagram. All patients were treated according to standard institutional anesthetic protocols that included premedication with fentanyl and midazolam and induction and maintenance of anesthesia with propofol, cisatracurium, and fentanyl according to standard intraoperative monitoring procedures.

**Fig. 2:**
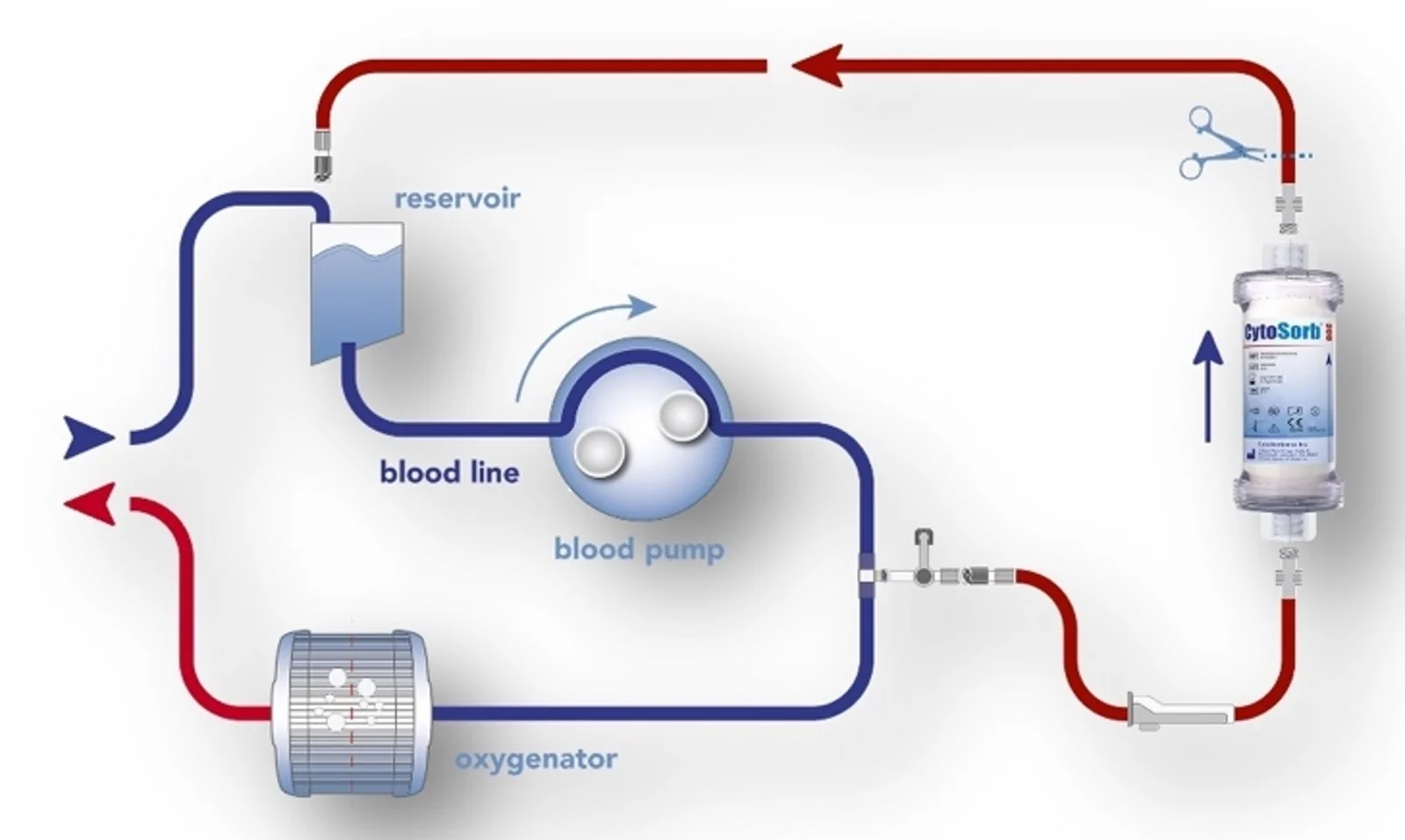
CytoSorb integration into cardio-pulmonary bypass

CABG was performed by the same surgical team according to standard on pump protocols. Heparin was administered for anticoagulation, targeting an activated clotting time (ACT) of over 480 s (300 IU of heparin per kilogram of body weight), and this was monitored every 30–45 min. Following discontinuation of CPB, complete reversal (1:1) was achieved using protamine sulphate, with an intended ACT ranging from 10% of baseline. Surgery was carried out using a conventional cardiopulmonary bypass (CPB) machine. In the intervention group, the device was integrated into the CPB circuit and remained active for the entire duration of CPB at a flow rate between 250 and 400 mL/min. (Fig. 2). The device was active during the entire CPB-run (CPB-time equals device time) without prolonging CPB-time. Following discontinuation of CPB, heparin was reversed with protamine sulfate and ACT restored to normal values. All patients were transferred to the intensive care unit while still intubated and were subsequently extubated once they met standard weaning criteria.

## Management of postoperative hemostasis and transfusion practices

Postoperative chest tube drainage (CTD) was recorded hourly for 24 h post-operatively. All patients included into the present analysis were treated by the same institutional transfusion protocol. Blood transfusions were administered when hematocrit levels fell below 24% (hemoglobin 8 g/dL), or when clinical signs indicated inadequate oxygen delivery as determined by the treating attending physicians. Platelets and fresh frozen plasma were transfused in cases of active bleeding, according to the *Guidelines on Patient Blood Management for Adult Cardiac Surgery* [24, 25]. Given that clopidogrel was the main contributor to coagulopathy in this cohort, platelet transfusion was prioritized, with FFP administered in response to abnormal coagulation parameters.

## Outcomes measures

The key outcomes measured in the study included 24-hour CTD, blood product transfusions, re-operations for bleeding and in-hospital mortality. Bleeding was also evaluated according to the standardized Bleeding Academic Research Consortium (BARC) definition and specifically BARC grade 4 bleeding that captures severe bleeding after CABG. BARC-4 bleeding is defined as: intracranial bleeding within 48 h, or re-operation for bleeding control, transfusion of 5 or more units of whole blood or packed red blood cells (pRBC) within 48 h, or 24-hr CTD > 2000mL Safety of the device was assessed by investigator-reported adverse events including severity and related classifications.

### Statistical analysis

Data were analyzed using the SAS software package, version 9.4 (SAS Institute, Cary, NC, USA). Continuous variables were expressed as mean with standard deviation (SD) or median with interquartile range (IQR), respectively, and compared using Student’s t-test or the Mann-Whitney U test when the requirements of normal distribution for t-test were not met. Categorical data were expressed as number and frequencies and compared using the chi-square test or Fisher´s exact test.

Since endpoints “reoperation” “and BARC-4” showed only one single event, odds ratios (OR) and 95%CI (confidence intervals) were calculated based on the Yates’s correction for continuity method. P-values < 0.05 were considered statistically significant. In addition to p-values, effect sizes were calculated to quantify the magnitude of differences between groups and to facilitate interpretation of the clinical relevance of the findings.

## Results

A total of 50 patients (mean age of 64.0 ± 8.6 years; 41 males and 9 females) undergoing on-pump CABG within 24 h of clopidogrel discontinuation were enrolled in the study. Patients were randomly assigned into Group 1 (intervention) or Group 2 (control). As shown in Table 1, there were no differences between the two groups in terms of demographics.

**Table 1:** Demographics

Variable	Group 1 *n* = 25	Group 2 *n* = 25
Age, years	62.9 ± 7.6	65.1 ± 9.5
Gender, male	19 (76)	22 (88)
Weight, kg	77.2 ± 11.4	78.7 ± 17.9
Diabetes	8 (32)	13 (52)
Myocardial infarction	4 (16)	3 (12)
Systemic hypertension	18 (72)	13 (52)
Ejection fraction, %	49.4 ± 6.7	42.3 ± 8.6

Data presented as mean±standard deviation or numbers (%)

Table 2 summarizes the intra- and postoperative outcomes, including those related to bleeding. Patients in Group 1 showed similar CPB and aortic cross-clamping times (median 66 (IQR: 56–75) vs. 75 (IQR: 60–93) minutes, *p* = 0.129 and 37 (IQR: 31–51) vs. 43 (IQR: 35–52) minutes, *p* = 0.459, respectively). Both groups were comparable in terms of BARC-4 bleeding events. No patients from either group had more than 2 L of CTD within 24 h or received more than five pRBCs over 48 h. In the control group (Group 2), one patient underwent surgical revision for bleeding control, counting as a single BARC-4 bleeding event. Nevertheless, Group 1 exhibited significantly less CTD during the first 24 h postoperatively (median 24-hr CTD: 300mL [250–400] vs. 500mL [400–700], *p* = 0.0003). Furthermore, patients in Group 1 required significantly fewer transfusions of pRBC, platelets, or fresh frozen plasma, as shown in Table 2. Moreover, device use was assessed as easy and safe by all perfusionists without any device-related adverse events reported. Of note, no hemolysis, thrombocytopenia or adsorber clogging was observed. Table 2: Intra- and postoperative operative outcomes.

**Table 2:** Intra- and postoperative outcome parameters

Indexes	Group 1 (*n* = 25)	Group 2 (*n* = 25)	Effect size	*P*-value
CPB time (min)	68.4 ± 16.4 66 (56, 75)	73.8 ± 21.2 75 (60, 93)	Mean difference: 95%CI -9.6 (-22.1, 2.9)	0.129
Clamp time (min)	41.5 ± 12.3 37 (31, 51)	44.7 ± 17.0 43 (35, 52)	Mean difference: 95%CI -3.2 (-11.9, 5.5)	0.459
ACT after surgery (s)	122.5 ± 4.4 123 (119, 126)	123.3 ± 3.5 123.5 (121, 126)	Mean difference: 95%CI -0.8 (-3.2, 1.6)	0.503
24-hr CTD (mL)	332 ± 189 300 (250, 400)	586 ± 285 500 (400, 700)	Mean difference: 95%CI -254 (-383, -125)	0.0003
RBC transfusions in first 24 h (units)	0.5 ± 0.7 0 (0, 1)	1.2 ± 1.2 1 (0, 2)	Non-normal distribution	0.047
PLT transfusions in first 24 h (units)	0.4 ± 1.3 0 (0, 0)	2.1 ± 1.4 2 (1, 3)	Non-normal distribution	< 0.0001
FFP transfusions in first 24 h (units)	0.16 ± 0.8 0 (0, 0)	2.1 ± 1.4 2 (2, 3)	Non-normal distribution	< 0.0001
Intracranial bleeding	0 (0)	0 (0)	N/A	1.00
> 5 pRBC/48 hours	0 (0)	0 (0)	N/A	1.00
> 2 L CTD / 24 h	0 (0)	0 (0)	N/A	1.00
Reoperations, n (%)	0 (0.0%)	1 (4.0%)	OR: 0.33 (0.01, 8.57) (based on Yates’s correction for continuity method)	0.487
BARC-4 bleeding	0 (0.0%)	1 (4.0%)	OR: 0.33 (0.01, 8.57) (based on Yates’s correction for continuity method)	0.487
In-Hospital mortality	0 (0)	0 (0)	N/A	1.00

Data presented as mean±standard deviation and median and IQR. Cardiopulmonary Bypass (CPB), Activated Clotting Time (ACT), Pack Red blood Cell (pRBC), Hour (hr), Chest Tube drainage (CTD), Platelets (PLT), Fresh Frozen Plasma (FFP)

## Discussion

The present randomized, controlled study aimed to evaluate the efficacy of intraoperative hemoadsorption in reducing perioperative bleeding in patients on clopidogrel undergoing urgent CABG within 24 h of drug discontinuation. The main observation of the study is that device use was associated with significant reductions in 24-hr CTD volume and blood product transfusions.

One of the major challenges in ACS patients undergoing urgent CABG surgery is achieving a balance between effective antiplatelet therapy and minimizing the risk of postoperative bleeding. Several studies have demonstrated that the residual antiplatelet effects of clopidogrel - particularly when surgery is performed within 5 to 7 days of the last dose - significantly increases the risk of perioperative bleeding and the need for blood product transfusion [26, 27].

Current guidelines recommend delaying surgery for at least five days after discontinuing clopidogrel to reduce the risk of excessive bleeding and improve surgical outcomes [25]. However, in non-deferable, urgent clinical settings, adherence to this waiting period is often not feasible. Given that the guideline-recommended washout period for clopidogrel is 5 days, all patients in our cohort underwent surgery substantially earlier than the recommended interval, and therefore significant residual antiplatelet activity can reasonably be assumed.

Clopidogrel is a prodrug that undergoes hepatic biotransformation to produce an active thiol metabolite. This metabolite then covalently binds to a cysteine residue on the extracellular domain of the platelet P2Y_12_ receptor, a key component in platelet activation and aggregation [28]. The covalent alkylation of cysteine residues is a highly specific and irreversible process, driven by the strong affinity of the thiol group for nucleophilic centers on the receptor surface. This interaction results in permanent inactivation of the receptor for the lifespan of the affected platelets, which typically ranges from 7 to 10 days.

Due to this irreversible binding, the antiplatelet effect of clopidogrel cannot be reversed pharmacologically and persists until new platelets are generated [28, 29]. Transfusion of fresh platelets is an option for clopidogrel treated patients since the active drug is irreversible bound on existing platelets, however, platelet reactivity may still not fully improve. Interestingly, in the present trial, intraoperative hemoadsorption resulted in significantly fewer platelet transfusions.

Hemoadsorption operates on the principle of physical adsorption, whereby solutes are removed from blood via transient, non-specific interactions with a porous sorbent material [30]. The efficiency of this process depends on several factors, including the physicochemical properties of the substance, the composition and surface area of the adsorbent, blood flow rate, and saturation kinetics. Commonly used adsorbents include macroporous resins, activated charcoal, and advanced polymer-based matrices such as CytoSorb [31]. These materials exhibit broad affinity for various molecules through multiple mechanisms, including van der Waals forces, hydrophobic interactions, hydrogen bonding, and π-π stacking [13, 32].

Clopidogrel, a hydrophobic compound with a molecular weight of 321.82 Da, is well within the adsorptive range of CytoSorb adsorbers. Both clopidogrel and its active metabolite are approximately 94%-98% protein-bound in plasma, leaving only a small fraction of free (unbound) drug capable of binding to platelet receptors [29]. The active metabolite binds irreversibly to platelets and theoretically may not be available for removal. As such, the exact mechanism underlying the bleeding reductions observed in the current study is not entirely clear and only hypothetical, although it may be related to removal of any remaining circulating free fraction of clopidogrel or its active metabolite since device exposure occurred within 24 h of the last dose.

Recent studies have highlighted hemoadsorption as a promising approach for the intraoperative removal of ticagrelor during open-heart surgery, demonstrating a reduction in postoperative bleeding as a result [32–35]. Given that ticagrelor is also a P2Y_12_ receptor antagonist, our findings align with these reports and further support the concept that hemoadsorption is effective in removing antiplatelet agents from the circulation [36]. It is important to note, however, that ticagrelor’s reversible binding to the P2Y_12_ receptor likely enhances the efficiency of its removal via hemoadsorption [34]. In contrast to ticagrelor, clopidogrel’s active metabolite binds irreversibly to platelet receptors, limiting the scope of removal to only the unbound fraction in plasma. Nevertheless, by potentially reducing remaining circulating levels of clopidogrel, hemoadsorption might contribute to improved postoperative hemostasis in theory only without any mechanistic evidence so far.

In contrast to the present study, Hassan et al. demonstrated a significant 67.1% reduction in ticagrelor plasma concentrations between the start and end of cardiopulmonary bypass. Accordingly, their findings provide direct mechanistic evidence supporting the ability of hemoadsorption to remove ticagrelor from the circulation. In comparison, the present study did not include measurements of clopidogrel plasma concentrations or platelet function, thereby limiting mechanistic interpretation of the observed clinical effects [34].

Moreover, the risk of bleeding is not solely dependent on the presence of antithrombotic agents but is also influenced by patient-specific factors such as hepatic and renal function, comorbid conditions, as well as duration and complexity of the surgical procedure itself. Given that most patients with ischemic heart disease receive dual antiplatelet therapy, the clinical population undergoing cardiac surgery is increasingly at elevated risk for postoperative bleeding. In such high-risk individuals, even small alterations in antiplatelet activity can lead to significant changes in bleeding risk [26]. In the current study plasma clopidogrel levels or platelet function were not measured, however, the clinical evidence of device efficacy was only based on clinically meaningful reductions in chest tube drainage and postoperative transfusion requirements.

An interesting observation of the present study is that postoperative coagulation parameters and platelet counts were broadly comparable between groups despite lower bleeding rates and reduced transfusion requirements in the hemoadsorption group. Specifically, patients treated with hemoadsorption received fewer platelet and red blood cell transfusions, yet postoperative platelet counts remained similar between groups. However, given the small sample size and the absence of platelet function testing or measurements of clopidogrel plasma concentrations, the clinical significance of these laboratory findings remains uncertain.

From a clinical perspective, the use of hemoadsorption in patients undergoing urgent or emergent surgery may be particularly beneficial in cases of recent clopidogrel intake, where a significant proportion of the drug remains unbound in plasma. Therefore, early initiation of hemoadsorption may help reduce bleeding risk by removing residual active compound before it irreversibly binds to platelet receptors. However, once clopidogrel has been systemically distributed and its active metabolite has bound to platelets, the effectiveness of extracorporeal removal strategies such as hemoadsorption will likely be diminished [2, 37].

Overall, the present findings suggest that intraoperative hemoadsorption is a potential strategy that may help to potentially mitigate postoperative bleeding in patients on clopidogrel undergoing urgent CABG. The current observations are comparable with previous studies evaluating intraoperative hemoadsorption in patients treated with ticagrelor [33, 34]. However, ticagrelor exhibits fundamentally different pharmacodynamic properties compared to clopidogrel. The ongoing, international Safe and Timely Antithrombotic Removal (STAR) Registry is currently capturing clinical usage patterns and associated clinical outcomes with the use of intraoperative hemoperfusion in patients on antithrombotic medications undergoing cardiac surgery [38, 39]. Hemoadsorption for antithrombotic drug removal is a relatively new technology, and data regarding its long-term clinical effects and safety remain limited.

## Limitations

The present study has certain limitations. Firstly, as a single center study with a relatively small sample size the generalizability of the findings may be limited. Secondly, due to international sanctions affecting our country, we were unable to measure clopidogrel plasma concentrations before and after hemoadsorption, nor could we perform dedicated platelet function testing. This limited our ability to elucidate the exact mechanism underlying the clinical observations. However, the patient population in the current trial included individuals requiring CABG surgery within 24 h of clopidogrel discontinuation; in other words, urgent surgery that could not be delayed allowing for a longer washout period. In such patients, platelet function testing does not inform or alter the course of care. It is worth noting that platelet function is altered after CPB, resulting in deficient platelet reactivity. Therefore, any proposed mechanisms underlying the observed clinical associations remain speculative, and the potential influence of unmeasured confounders cannot be excluded. Thirdly, the present findings are limited to patients undergoing urgent CABG within 24 h of clopidogrel discontinuation and may not be generalizable to patients who complete the recommended preoperative washout period. Finally, although transfusion management followed a predefined institutional protocol, the potential influence of transfusion practices on the observed results cannot be excluded.

## Conclusion

While the irreversible binding of clopidogrel to the P2Y_12_ receptor may suggest limited efficacy of hemoadsorption in removing the drug from circulation, that current findings demonstrate that this intraoperative device use might reduce postoperative blood loss and the need for blood product transfusions in patients on clopidogrel undergoing urgent CABG surgery. Accordingly, hemoadsorption may be considered as a valuable adjunct in blood management protocols, particularly for high-risk surgical cases. As the underlying mechanisms are still unclear, it must be acknowledged that the clinical associations are speculative. Although the present findings suggest a potential benefit of intraoperative hemoadsorption in clopidogrel-treated patients undergoing urgent CABG, the single-center design and relatively small sample size limit the generalizability of the results and warrant confirmation in larger multi-center studies. Further studies are required to validate these observations and elucidate the exact mechanisms underlying the observed benefits.

**Table 3:** Coagulation and laboratory parameters

		Group 1 *n* = 25	Group 2 *n* = 25
PT (s)	Before surgery	14.7 ± 4.9	12.6 ± 1.6
	After surgery	17.8 ± 9.9	13.3 ± 1.4
PTT (s)	Before surgery	34.7 ± 6.6	30.1 ± 8.5
	After surgery	46.9 ± 10.2	53.2 ± 30.0
INR	Before surgery	1.2 ± 0.4	1.1 ± 0.2
	After surgery	1.5 ± 0.9	1.2 ± 0.1
HB (g/dL)	Before surgery	13.7 ± 1.6	13.1 ± 1.7
	After surgery	10.0 ± 1.1	11.2 ± 1.3
HCT (Ratio)	Before surgery	41.7 ± 4.6	39.8 ± 4.2
	After surgery	31.5 ± 3.6	33.5 ± 4.4
PLT (1000/µL)	Before surgery	194.6 ± 52.5	216.8 ± 54.3
	After surgery	185.5 ± 48.8	190.4 ± 43.2
BUN (mg/dL)	Before surgery	26.7 ± 15.7	34.2 ± 8.5
	After surgery	43.5 ± 9.3	41.3 ± 8.3
Cr (mg/dL)	Before surgery	1.3 ± 0.3	1.2 ± 0.2
	After surgery	1.3 ± 0.5	1.2 ± 0.2
ALT (U/L)	Before surgery	21.2 ± 7.7	25.8 ± 9.1
	After surgery	26.8 ± 11.0	27.3 ± 11.0
AST (U/L)	Before surgery	30.3 ± 13.5	29.0 ± 8.9
	After surgery	59.5 ± 38.1	49.7 ± 14.2
LDH (U/L)	Before surgery	349.3 ± 110.0	332.8 ± 112.0
	After surgery	642.2 ± 228.9	560.7 ± 134.2

Data presented as mean±standard deviation. Prothrombin time (PT), Partial thromboplastin time (PTT), International normalized ratio (INR), Hemoglobin (Hb), Hematocrit (HCT), platelet (PLT), Blood urea nitrogen (BUN), Creatine (Cr), Alanine transaminase (ALT), AST (aspartate aminotransferase), Lactate dehydrogenase (LDH)

## Data Availability

All data will be provided upon request by the corresponding author.

## Abbreviations

ACS: Acute coronary syndrome
ACT: Activated clotting time
ADP: Adenosine diphosphate
ALT: Alanine transaminase
ASA: Aspirin
AST: Aspartate aminotransferase
BARC: Bleeding academic research consortium
BUN: Blood urea nitrogen
CABG: Coronary artery bypass grafting
CI: Confidence interval
CPB: Cardiopulmonary bypass
Cr: Creatinine
CTD: Chest tube drainage
DAPT: Dual antiplatelet therapy
DOAC: Direct oral anticoagulant
ECMO: Extracorporeal membrane oxygenation
FFP: Fresh frozen plasma
Hb: Hemoglobin
HCT: Hematocrit
IABP: Intra-aortic balloon pump
ICU: Intensive care unit
INR: International normalized ration
IQR: Interquartile range
LDH: Lactate dehydrogenase
LVEF: Left ventricular ejection fraction
OR: Odds ratio
pRBC: packed red blood cells
PLT: Platelets
PPT: Partial prothrombin time
PT: Prothrombin time
PVD: Peripheral vascular disease
SD: Standard deviation
